# Boosted Pro-Inflammatory Activity in Human PBMCs by Lipopolysaccharide and SARS-CoV-2 Spike Protein Is Regulated by α-1 Antitrypsin †

**DOI:** 10.3390/ijms22157941

**Published:** 2021-07-26

**Authors:** Srinu Tumpara, Anna R. Gründing, Kokilavani Sivaraman, Sabine Wrenger, Beata Olejnicka, Tobias Welte, Maria J. Wurm, Paco Pino, Divor Kiseljak, Florian M. Wurm, Sabina Janciauskiene

**Affiliations:** 1 Department of Respiratory Medicine, Member of the German Center for Lung Research (DZL), Biomedical Research in Endstage and Obstructive Lung Disease Hannover (BREATH), Hannover Medical School, 30625 Hannover, Germany; Tumpara.Srinu@mh-hannover.de (S.T.); Anna.R.Gruending@stud.mh-hannover.de (A.R.G.); Sivaraman.Kokilavani@mh-hannover.de (K.S.); Wrenger.Sabine@mh-hannover.de (S.W.); beata.olejnicka@med.lu.se (B.O.); Welte.Tobias@mh-hannover.de (T.W.); 2 ExcellGene SA, 1970 Monthey, Switzerland; Maria.Wurm@excellgene.com (M.J.W.); Paco.Pino@excellgene.com (P.P.); Florian.Wurm@excellgene.com (F.M.W.); 3 École Polytechnique Fédérale de Lausanne, Faculty of Life Sciences, 1015 Lausanne, Switzerland; Divor.Kiseljak@excellgene.com

**Keywords:** COVID-19, spike, LPS, AAT, PBMCs, inflammation, cytokines, chemokines

## Abstract

For the treatment of severe COVID-19, supplementation with human plasma-purified α-1 antitrypsin (AAT) to patients is currently considered. AAT inhibits host proteases that facilitate viral entry and possesses broad anti-inflammatory and immunomodulatory activities. Researchers have demonstrated that an interaction between SARS-CoV-2 spike protein (S) and lipopolysaccharides (LPS) enhances pro-inflammatory responses in vitro and in vivo. Hence, we wanted to understand the potential anti-inflammatory activities of plasma-derived and recombinant AAT (recAAT) in a model of human total peripheral blood mononuclear cells (PBMCs) exposed to a combination of CHO expressed trimeric spike protein and LPS, ex vivo. We confirmed that cytokine production was enhanced in PBMCs within six hours when low levels of LPS were combined with purified spike proteins (“spike”). In the presence of 0.5 mg/mL recAAT, however, LPS/spike-induced TNF-α and IL-1β mRNA expression and protein release were significantly inhibited (by about 46–50%) relative to LPS/spike alone. Although without statistical significance, recAAT also reduced production of IL-6 and IL-8. Notably, under the same experimental conditions, the plasma-derived AAT preparation Respreeza (used in native and oxidized forms) did not show significant effects. Our findings imply that an early pro-inflammatory activation of human PBMCs is better controlled by the recombinant version of AAT than the human plasma-derived AAT used here. Considering the increasing clinical interest in AAT therapy as useful to ameliorate the hyper-inflammation seen during COVID-19 infection, different AAT preparations require careful evaluation.

## 1. Introduction

Coronavirus disease 2019 (COVID-19) is characterized by hyper-inflammation and coagulopathy, and in severe cases, an emerging “cytokine storm” can lead to respiratory failure, the onset of sepsis, and death. However, the triggers initiating excessive inflammation and abnormally strong immune responses during SARS-CoV-2 infection remain largely unknown. Consequently, the inhibition of a cytokine storm is one of the main strategies in the treatment of severely ill SARS-CoV-2 patients. Current therapies include non-specific antiviral drugs, antibiotics used for the treatment of secondary bacterial infections, and anti-inflammatory preparations, including NSAIDs, glucocorticoids, and antagonists of specific pro-inflammatory cytokines [1]. However, each approach has apparent limitations either because of restricted anti-inflammatory effects or because of undiscriminating suppression of all pro- and anti-inflammatory mediators [2]. At present, an established treatment for patients with COVID-19 remains unavailable. Thereby, for finding targeted pharmacological treatments, a comprehensive understanding of processes underlying SARS-Cov-2 infectivity remains of critical importance.

SARS-CoV-2 is a single-stranded RNA-enveloped virus covered by many trimeric class I fusion spike (S) glycoproteins [3]. The monomeric spike molecule of SARS-CoV-2 is 1273 amino acid-long that contains a signal peptide and two subunits, denoted as S1 and S2, which can undergo large structural rearrangements to fuse the viral and host cell membranes [4]. Because the trimeric spike structure (spike) is involved in receptor recognition, viral attachment, and entry into host cells, it represents one of the most important targets for COVID-19 diagnostics, vaccine and therapeutic development, and research [5].

During a SARS-CoV-2 infection, the interplay between angiotensin-converting enzyme 2 (ACE2), desintegrin and metalloproteinase domain 17 (ADAM17), and type II transmembrane serine protease (TMPRSS2) is an important factor. While TMPRSS2 cleaves the monomeric spike molecules into the aforementioned S1 and S2 subunits allowing virus entry [6], the spike/ACE2 interaction may trigger ADAM17 activation, and ADAM17-mediated ACE2 cleavage further intensifies virus entry and harmful effects [7,8]. Several studies indicated that human α1-antitrypsin (AAT, encoded by SERPINA1 gene) inhibits the activities of both TMPRSS2 and ADAM17 [9,10]. Concomitantly, AAT was found to decrease SARS-CoV-2 copy numbers within target cells [11].

AAT is an acute phase glycoprotein mainly produced by liver cells but also by monocytes/macrophages. In healthy persons, its concentrations in blood circulation range from 0.9 to 2 g/L, whereas in response to inflammation, AAT levels can increase very fast between four- and six-fold [12]. In addition to strong inhibitory activity against serine proteases, AAT can scavenge free radicals and free heme and regulates the expression and release of cytokines/chemokines [13,14,15]. Recently, we demonstrated that physiological concentrations of AAT quickly inhibit ATP-induced release of IL-1β from primary human blood mononuclear cells and rat lung tissue. Interestingly, this anti-inflammatory function of AAT was independent of its anti-protease activity [16]. In animal models, therapy with AAT protected from ischemia-reperfusion injury, rejection islet allografts, graft-versus-host-disease, rheumatoid arthritis, TNF-α/endotoxin induced lethality, and sepsis [17,18,19,20]. In general, one can consider AAT as an immunomodulatory protein expressing anti-inflammatory and anti-protease activities, which can act independently of each other [21].

A recent study demonstrated an interaction between SARS-CoV-2 spike protein and lipopolysaccharide (LPS), an established TLR4 ligand, boosting pro-inflammatory cell responses in vitro and in vivo [22]. The observed synergism between LPS and spike protein gave new insights into risk of severe COVID-19 disease and encourages a search for potentially fast-acting anti-inflammatory pharmaceuticals. We thought that human plasma-derived and/or a recombinant CHO produced AAT [23,24], might be useful candidates to test if they can prevent or reduce LPS/spike-induced activation of human PBMCs ex vivo.

## 2. Results

### 2.1. Characterization of AAT Proteins

**Figure 1:**
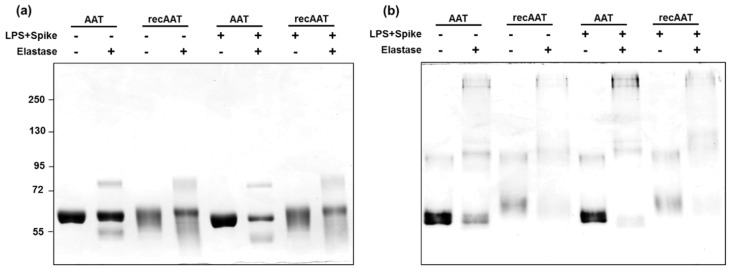
Characterization of plasma-derived AAT and recombinant AAT by elastase complex formation: AAT (0.5 mg/mL) protein was mixed with porcine pancreas elastase in a molar ratio of 2:1 (AAT/elastase). In some cases, 10 ng/mL LPS and 12 µg/mL spike was added. Samples were incubated for 30 min at room temperature. (**a**) Proteins were separated on a 7.5% polyacrylamide SDS gel and (**b**) on a 7.5% native gel in the absence of SDS under non-reducing conditions. The gels were stained with Coomassie. The figure shows representative gels from two independent experiments.

Both recombinant AAT (ExcellGene SA, Switzerland) and plasma-derived AAT (Respreeza, CSL Behring) were prepared in sterile PBS. We characterized proteins qualitatively and checked for their anti-elastase activities in vitro and in the supernatants of human PBMCs incubated for 6 h with proteins alone or in combination with 10 ng/mL LPS or LPS plus 12 µg/mL spike. All in vitro prepared samples were analyzed on 7.5% SDS-PAGE or native 7.5% PAGE followed by staining with Coomassie brilliant blue (Figure 1a,b).

The results identified a major band of about 60 kDa for native and recAAT which was compatible with the expected mass due to AAT glycosylation. Both proteins formed complexes with elastase independently of the presence of LPS or LPS/spike (Figure 1a, AAT/elastase complex, about 72 kDa). Notably, recAAT produced a smear on SDS-PAGE, indicating heterogeneity in glycosylation.

The same samples were also examined in 7.5% native-PAGE with an aim to separate AAT proteins according to the net charge, size, and shape of their native structure. As illustrated in Figure 1b, the profiles of the two proteins were similar. However, recAAT migrated more slowly than plasma-derived AAT, implying a higher positive charge density in recAAT (more charges per molecule mass).

**Figure 2:**
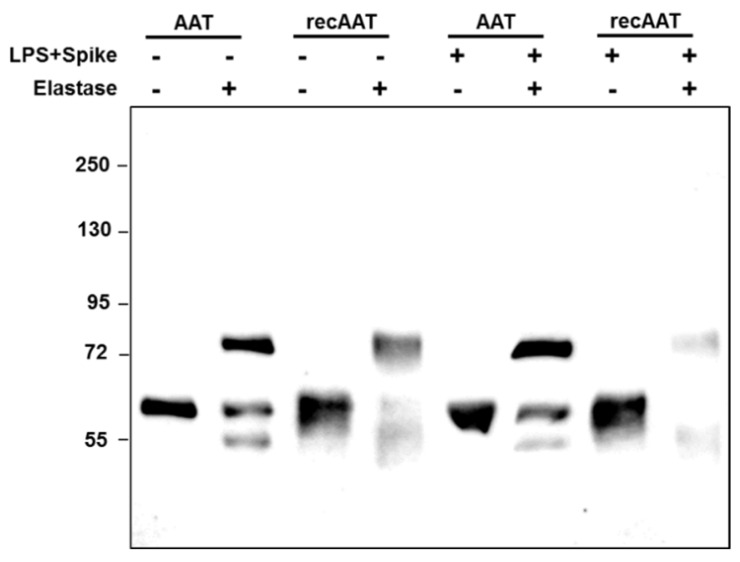
Analysis of AAT elastase complex formation in culture supernatants. PBMCs were incubated with 0.5 mg/mL plasma-derived AAT or recombinant AAT. Some cells were cultured with a combination of AAT proteins together with 10 ng/mL LPS and 12 µg/mL spike. After 6 h of incubation at 37 °C, cultured supernatants were harvested. The supernatants were further incubated with PBS or porcine pancreas elastase in a molar ration of 2:1 (AAT/elastase) for 30 min at room temperature. The complex formation was stopped by addition of SDS sample buffer and heating at 90 °C for 2 min and analyzed by Western blotting. AAT and AAT-containing complexes were targeted with rabbit polyclonal anti-AAT antibody and anti-rabbit-horseradish peroxidase and visualized with ECL Western blotting substrate. The figure shows one representative blot out of two independent experiments.

The Western blots of supernatants collected after 6 h of PBMCs culture revealed again that both AAT proteins independently of the presence of LPS or LPS/spike did not change molecular sizes and formed complexes with elastase (Figure 2).

### 2.2. Effects of AAT Proteins on Cytokine/Chemokine Expression

Next, we assessed whether AAT proteins (0.5 mg/mL) had any effect on cytokine/chemokine expression when added to total PBMCs for 6 h in the presence of 10 ng/mL LPS or LPS plus 12 µg/mL spike (LPS/spike). In this study, we focused on TNFα, IL-1β, and IL-6 as known pro-inflammatory markers of acute inflammatory response, along with CXCL8/IL-8 because of its potent role in the recruitment and activation of neutrophils, commonly elevated in patients with COVID-19 [25]. These cytokines have also been suggested as biomarkers to predict severity and survival of COVID-19 patients [26]. As expected, LPS alone and LPS in combination with spike protein caused a significant induction in mRNA expression of all analyzed markers (Table 1). Surprisingly, only when PBMCs were co-cultured for 6 h with LPS/spike and recAAT, a significant reduction in *TNFA* and *IL1B* mRNA was observed as compared to cells stimulated with LPS/spike alone. Although without statistical significance, recAAT also reduced LPS/spike-induced *IL6* mRNA (by about 50%) and slightly lowered *CXCL8* mRNA (by about 24%). As shown in Table 1, under the same experimental conditions, plasma AAT in its native or oxidized form had no effect on cytokine/chemokine expression. It is of importance to point out that spike protein as well as all AAT preparations, when added to PBMCs alone for 6 h, showed no significant effect on the expression of the analyzed markers (data not shown).

### 2.3. Effects of AAT Proteins on Cytokine/Chemokine Release after 6 Hours of PBMCs Culture

In parallel to gene expression analysis, we also measured cytokine/chemokine release after PBMCs culture for 6 h in the presence of 10 ng/mL LPS or LPS plus 12 µg/mL spike with and without 0.5 mg/mL of AAT preparations. As shown in Table 2, both LPS and LPS/spike strongly induced all pro-inflammatory cytokine/chemokine release. The co-stimulation with LPS/spike resulted in about 40% higher TNF-α secretion as compared to that with LPS alone, whereas release of other measured markers was only minimally enhanced. Again, in concordance with gene expression data, addition of recAAT to LPS/spike-treated PBMCs strongly lowered the release of TNF-α- and IL-1β and, to a lesser extent, IL-6 and IL-8 as compared to LPS/spike-treated cells, whereas native AAT had no effect. Noticeably, LPS/spike-induced IL-1β release was slightly lowered (by about 25%) in the presence of plasma oxAAT.

## 3. Discussion

The analysis of the immune characteristics of patients with severe COVID-19 revealed significantly upregulated levels of cytokines, including IL-6, TNF-α, IL-1β, and IL-8, among others. Some authors suggested that specifically IL-6 is the key cytokine leading to inflammatory storm phenomena increasing alveolar-capillary blood-gas exchange dysfunction, pulmonary fibrosis, and organ failure [27]. Therefore, drugs which can interfere immediately with pro-inflammatory cytokines might be potential therapeutics for severe and critical COVID-19. Human plasma AAT preparations attract the attention of clinicians as potential therapy for COVID-19 patients. There are at least two reasons for this: first, AAT is an acute phase protein expressing broad anti-inflammatory activities and already proven as safe therapy in patients with inherited AAT deficiency. In support, we recently reported that exogenously given AAT provides significant lung protective immunity in AAT-deficient (KO) mice challenged with *Streptococcus pneumoniae* [28]. Secondly, although AAT increases in COVID-19 patients, the ratio of cytokines/AAT seems to be higher in patients with the severe/critical disease than in mild/stable disease and is associated with a poor outcome [2]. Therefore, AAT augmentation therapy is considered useful to modulate the production of key pro-inflammatory cytokines [13,29].

Several clinical reports demonstrated significantly increased levels of endotoxins in the plasma of severely affected COVID-19 patients [30,31]. Indeed, COVID-19 is not the only viral infection for which severity of disease is connected to the circulating LPS levels. For example, LPS levels were also connected to systemic immune activation during HIV infections [32]. Similarly, in murine models, combinations of the virus with LPS were found to induce severe hyper-cytokinesis in the lungs and a massive death rate of infected animals, although a single viral or LPS infection at the same doses did not demonstrate such outcomes [33]. Current data suggest that SARS-CoV-2 spike may act like superantigens and directly activate immune cells [34,35,36].

**Figure 3:**
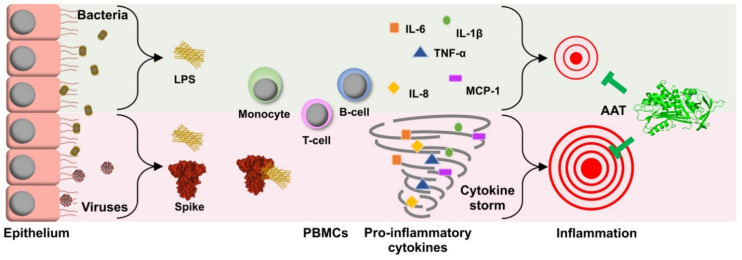
Schematic representation of putative suppression of the cytokine storm induced in the presence of SARS-CoV2 spike protein by alpha 1-antitrypsin (AAT): co-incubations of trimeric spike (S) protein along with bacterial LPS induced the pro-inflammatory cytokines production to several folds higher than in the presence of LPS alone. AAT is well-appreciated for its contribution in modulating the immune responses by suppressing pro-inflammatory cytokine and chemokine production. In addition, emerging evidence strongly suggests a role of AAT in the suppression of SARS-CoV2 infection. Our findings support a notion that AAT could dampen the cytokine storm caused by LPS-spike co-action.

Therefore, in this study, we aimed to address the question whether recombinant and plasma AAT preparations can, in a fast manner (within six hours), affect LPS/spike-induced pro-inflammatory cytokine/chemokine expression and release from total human PBMCs. Previous in silico studies predicted that the spike proteins of SARS-CoV-2 not only bind to the ACE2 receptor but also to toll-like receptors (TLRs), especially TLR4, a receptor for LPS, and thus can strongly enhance inflammatory responses [37,38]. This LPS/spike synergism prompted us to investigate if the spike could quickly enhance the pro-inflammatory effects of LPS in human PBMCs. To observe any additive effect of the spike protein, we chose to stimulate cells with relatively low concentrations of LPS (10 ng/mL). Indeed, within six hours, the spike enhanced LPS-induced expression of *TNFA*, *IL1B*, *IL6*, and *CXCL8*, but only minimally affected cytokine/chemokine release. Within this short period of time, none of the AAT proteins used showed a significant effect on LPS-induced expression and release of analyzed markers (Table 1 and Table 2). It is of interest to point out that when performing experiments with different models, including human PBMCs, we have observed that AAT proteins within a short period of time (between two and six hours) either synergize or show no effect on LPS-induced pro-inflammatory cytokine/chemokine production [39]. In fact, LPS and AAT may produce different results depending on their used concentrations. For example, in a model of adherent human monocytes, we found that low concentrations of AAT (0.02–0.1 mg/mL) could increase cytokine levels as compared to physiological concentrations of AAT (0.5–1 mg/mL) and non-treated controls [40]. Altogether, our findings support a notion that AAT regulates inflammation by a delicate and intricate balance between pro- and anti-inflammatory cellular responses and inspire further detailed investigations in this direction (Figure 3).

A somewhat unexpected finding was that within a short period of time, only a recombinant form of AAT was able to suppress significantly LPS/spike-induced expression and release of measured cytokines/chemokines in our human PBMC model. Currently, we are not able to explain why recAAT was more potent in suppressing LPS/spike-induced cell activation. However, based on the large body of literature about human plasma AAT and the coronavirus spike proteins, we provide here a few speculations. The first is that AAT plays a biologically relevant role in restriction of tissue responses to pro-inflammatory stimuli via inhibition of various proteases but also via direct interactions with inflammatory substances and/or activated cells. Moreover, several studies have shown that AAT is internalized by various cells, including PBMC and endothelial cells [41,42,43,44]. For example, by performing more detailed microscopy studies on human endothelial cells, we recently found that internalized AAT is localized in lysosomes and prevents lysosomal alkalization (https://doi.org/10.1016/j.redox.2021.102060, accessed on 30 June 2021). Therefore, we cannot exclude that recAAT might have a faster interaction with cells and intracellular signaling pathways, although the two preparations studied here showed remarkably similar anti-elastase activity. The second is that biological activities of AAT are strongly influenced by the post-translational modifications of the AAT protein such as glycosylation, oxidation, polymerization, etc. [45]. For instance, the glycosylation of AAT is not only important in its anti-protease and immunomodulatory functions but also affects the half-life and prevents its aggregation [46]. The isoelectric focusing of AAT in the serum of severe COVID-19 patients showed highly sialylated glycoforms creating a negative charge of AAT protein [47]. This might indicate a specific host response to virus-induced inflammation and negatively affect biological functions of AAT. In fact, the spike-protein from SARS-CoV-2 was shown to bind sialic acids [48]. According to the native gel electrophoresis performed here, recAAT was more positively charged than plasma AAT, which might be at least one putative reason why recAAT was more efficient than plasma AAT at inhibiting LPS/spike-induced early activation of PBMCs. For example, native human plasma AAT possesses an N-glycan profile with ~60–80% diantennary, disialylated, non-fucosylated structures with human-like alpha-2,6-linked sialic acids. CHO cells lack active St6 beta galactoside alpha-2,6-sialyltransferase 1 (ST6GAL1) to cap N-glycans with alpha-2,6-linked sialic acids [49]. Therefore, recAAT N-glycosylation is far from the profile of plasma AAT [50]. Further studies to perform deep characterization of recAAT protein are in progress.

Use of plasma-derived AAT has been approved for decades, therefore, a rapid evaluation and repurposing of AAT as a potential drug for severe COVID-19 disease is possible [51,52]. Therapy with AAT could be valuable simultaneously in controlling viral burden as well as aberrant immune responses, especially in the case of increased risk in patients that become infected with bacteria [53]. It is, however, acknowledged that several critical questions still need to be answered and require deeper investigations on structural and functional interactions of virus spike proteins, AAT preparations, and bacterial endotoxins.

## 4. Materials and Methods

### 4.1. Protein Preparations

#### 4.1.1. Human Plasma Isolated AAT

Plasma-purified human AAT (99% purity, Respreeza, CSL Behring, Kankakee, IL, USA) was used for experiments after buffer exchange to the sterile phosphate buffered solution (PBS) (Gibco, Thermo Fisher Scientific, Waltham, MA, USA) by using 10 K MWCO centrifugal filter columns (Thermo Fisher Scientific).

#### 4.1.2. Human Plasma Oxidized AAT

Oxidized AAT was prepared from native plasma-purified AAT (see above) by adding N-chlorosuccinimide (Sigma-Aldrich, Merck, Darmstadt, Germany) to AAT solution at a molar ratio of 20:1. The mixture was allowed to react for 20 min at room temperature, and N-chlorosuccinimide was then removed by washing with PBS 3 times using viva spin-20 centrifugal columns with a 10 k MWCO (Sartorius, Göttingen, Germany).

#### 4.1.3. Recombinant AAT

A highly purified, glycosylated form of recombinant AAT (recAAT) was produced in Chinese hamster ovary (CHO) cells (ExcellGene, Monthey, Switzerland). AAT was buffer exchanged to the sterile phosphate buffered solution (PBS) (Gibco, Thermo Fisher Scientific, Waltham, MA, USA) by using 10 K MWCO centrifugal filter columns (Thermo Fisher Scientific) [24].

#### 4.1.4. Spike Protein

A highly purified secreted form of a trimeric spike protein was produced in CHO cells. Briefly, a mammalian expression vector based on the “Wuhan” version of the SARS-CoV-2 spike protein was constructed, which was modified by deletion of the transmembrane and the intracellular peptide sequences, as well by the inclusion of either 2 or 6 prolines to increase the stability of the pre-fusion form [54]. The internal furin-cleavage site sequence was mutated, and a T4-trimerisation domain was added, followed by His-tag sequence. A stable clonal cell line was scaled-up for production, and a fed-batch process was applied. Purified protein was obtained by affinity chromatography and a size-exclusion step [23].

### 4.2. Characterization of AAT Proteins by Elastase Complex Formation

For characterization of the biological function of AAT proteins, the formation of the AAT-elastase complex was assessed. AAT (0.5 mg/mL) was diluted by a factor of 1 in 25 and incubated with porcine pancreas elastase (Sigma-Aldrich, Merck, Darmstadt, Germany) in a molar ratio of 2:1 (AAT:elastase) for 30 min at room temperature. The complex formation was stopped by addition of SDS sample buffer. Samples were separated by 7.5% SDS-polyacrylamide gels. For native PAGE, 7.5% polyacrylamide gels, buffers without SDS were used. Gels were stained with Coomassie.

### 4.3. Isolation and Culture of Human PBMCs

Human PBMCs were isolated from healthy donor’s peripheral blood using Lymphosep (C-C-Pro, Oberdorla, Germany) by discontinuous gradient centrifugation according to the manufacturer’s instructions as described previously [55]. Cells from each donor were immediately suspended in RPMI-1640 media (Gibco, Thermo Fisher Scientific, Waltham, MA, USA) and plated into non-adherent 24-well plates (Greiner Bio-one, Kremsmünster, Austria) at a density of 2.5 ×10^6^ cells per well. Cell were incubated for 6 h at 37 °C and 5% CO_2_ with 10 ng/mL lipopolysaccharide (O111:B4) from *Escherichia coli* (Sigma-Aldrich, Merck, Darmstadt, Germany) alone or in combination with 12 µg/mL of highly purified, endotoxin-free recombinant spike trimer (S-protein) or co-stimulated with 0.5 mg/mL AAT proteins. Afterwards, the cells were used for RNA isolation or lysate preparation, and the supernatants were collected for cytokine/chemokine detection by ELISA.

### 4.4. Real-Time Polymerase Chain Reaction (RT-PCR) Analysis

Total RNA was prepared according to the manufacturer’s instructions by using the RNeasy Mini kit (Qiagen, Venlo, The Netherlands). RNA amounts were determined with the NanoDrop spectrophotometer (Thermo Fisher Scientific, Waltham, MA, USA). For cDNA synthesis, 1 µg total RNA was transcribed using a High Capacity cDNA Reverse Transcription Kit (Thermo Fisher Scientific). mRNA expression levels of selected genes were analyzed using TaqMan Gene Expression Assays (Thermo Fisher Scientific) on a StepOnePlus Real-Time PCR Systems machine (Thermo Fisher Scientific). All taqman primers were purchased from Thermo Fisher Scientific: *HPRT1* Hs01933259_m1; *CXCL8* Hs00174103_m1; *IL1B* Hs01555410_s1; *IL6* Hs00985639_m1; *TNFA* Hs00174128_m1. The Ct value for each sample was calculated by determining the point at which the fluorescence exceeded a threshold limit. *HPRT1* was used as a housekeeping gene in the same run. Basal expression of genes was calculated according to the method 2^∆Ct^ (Ct value of target gene—Ct value of reference gene). All measurements were performed in duplicates, with samples from seven independent donors.

### 4.5. Western Blot Analysis

By the end of incubation, cell-free supernatants were collected and complexed with elastase at 2:1 molar ratio for 30 min at room temperature. The reaction was terminated by addition of SDS sample buffer. The proteins were separated on 7.5% SDS-polyacrylamide gels prior to transfer onto polyvinylidene difluoride (PVDF) membranes (Millipore, Billerica, MA, USA). Membranes were blocked for 1 h with Tris-buffered saline containing 0.01% tween-20 and 5% low fat milk powder (Roth, Karlsruhe, Germany) and incubated at 4 °C with primary polyclonal rabbit anti-human AAT antibody (1:800) (DAKO, Glostrup, Denmark). The immune complexes were visualized with anti-rabbit horseradish peroxidase-conjugated secondary antibody (DAKO) and ECL Western blotting substrate (BioRad, Hercules, CA, USA). Images were taken by using a Chemidoc Touch imaging system (BioRad).

### 4.6. ELISA Assays

Cell-free culture supernatants were analyzed directly or stored at −80 °C. ELISA Duoset kits for TNF-α (assay detection range 15.6–1000 pg/mL), IL-1β (assay detection range 3.91–250 pg/mL), IL-6 (assay detection range 9.38–600 pg/mL), and CXCL8/IL-8 (assay detection range 31.3–2000 pg/mL) were purchased from R&D Systems, Minneapolis, MN, USA and used according to the manufacturer’s instructions. Plates were analyzed by using a microplate reader Infinite M200 (Tecan, Männedorf, Switzerland), measuring absorbance at 450 nm with the reference wavelength at 540 nm. All measurements were performed in duplicates with samples from seven independent donors.

### 4.7. Statistical Analysis

Data from qPCR and ELISA were statistically evaluated by the Friedman Repeated Measures Analysis of Variance on Ranks using Sigma Plot 14.0 software package (Systat Software GmbH, Erkrath, Germany). A p-value below 0.05 was considered as statistically significant. Data are presented as median and percentiles 25 and 75.

## Figures and Tables

**Table 1:** Gene expression in PBMCs at 6 h culture.

	Gene Expression (Relative to Housekeeping Gene *HPRT1*)							
Sample, *N* = 7 donors	*TNFA*		*IL1B*		*IL6*		*CXCL8*	
Control	**3.1**	(1.2–4.8)	43.2	(34.4–55.4)	0.07	(0.03–0.10)	75.6	(46.2–95.3)
LPS	31.5	(38.4–59.9)	559.2	(452.8–686.0)	63.0	(40.8–105.7)	965.5	(852.4–1394.4)
LPS + AAT	28.1	(8.3–63.0)	511.6	(490.8–625.3)	62.8	(43.7–95.6)	986.6	(809.5–1126.6)
LPS + oxAAT	28.6	(9.4–34.6)	574.2	(447.3–733.7)	47.7	(40.7–83.4)	1077.9	(833.5–1247.1)
LPS + recAAT	14.6	(8.0–32.7)	637.3	(489.4–725.4)	50.3	(35.5–68.0)	1208.9	(1001.2–1323.7)
LPS + Spike	33.7	(24.4–163.1)	853.2	(729.1–903.9)	110.4	(83.8–127.2)	1540.2	(1248.2–1983.4)
LPS + Spike + AAT	34.5	(25.7–109.7)	751.5	(639.2–903.6)	102.6	(56.5–112.7)	1735.2	(1319.5–1841.3)
LPS + Spike + oxAAT	33.3	(20.7–129.1)	668.4	(517.2–868.8)	104.6	(49.2–124.5)	1610.6	(846.7–2190.2)
LPS + Spike + recAAT	**16.8**	(13.4–38.8) *	**472.5**	(320–754.1) **	49.7	(32.3–73.8)	1183.9	(1012.7–1597.4)

Data presented as median (range), bold shows significant difference between LPS/spike and LPS/spike + recAAT, * *p* < 0.05, ** *p* < 0.01. All measurements were performed in duplicates, with samples from seven independent donors using 2.5 × 10^6^ cells per experimental condition.

**Table 2:** Cytokine/chemokine release in supernatants after PBMCs culture for 6 h.

	Cytokine/Chemokine Concentration (pg/mL Supernatant)							
Sample, *N* = 7 donors	TNF-α		IL-1β		IL-6		IL-8	
Control	12.4	(8.7–34.5)	4.9	(1.9–5.9)	2.3	(1.53–3.0)	167.0	(57.4–456.4)
LPS	1684	(1359–2762)	1271	(675–2204)	4844	(3330–7304)	18,323	(15,095–27,513)
LPS + AAT	1967	(1452–3050)	1582	(980–2501)	6944	(6118–11,008)	18,691	(16,899–25,970)
LPS + oxAAT	1926	(1390–3605)	1165	(729–1652)	9857	(6744–14,231)	21,283	(17,394–32,982)
LPS + recAAT	1555	(1225–2538)	1151	(491–1699)	6683	(4976–9377)	19,365	(16,595–32,256)
LPS + Spike	2326	(2078–3373)	1222	(697–1791)	5136	(3446–9070)	20,376	(14,231–27,923)
LPS + Spike + AAT	2103	(1531–2840)	1116	(685–1756)	5211	(3177–9524)	18,984	(14,155–25,301)
LPS + Spike + oxAAT	2575	(1736–4020)	922	(799–1585)	8041	(7184–11,013)	20,236	(17,831–32,659)
LPS + Spike + recAAT	**1266**	(984–1899) **	**505**	(252–916) *	4753	(2399–8200)	17,072	(14,359–21,857)

Data presented as median (range), bold shows significant difference between LPS/spike and LPS/spike + recAAT, * *p* < 0.05, ** *p* < 0.01. All measurements were performed in duplicates, with samples from seven independent donors using 2.5 × 10^6^ cells per experimental condition.
